# Tumor necrosis factor-α blockade treatment decreased CD154 (CD40-ligand) expression in rheumatoid arthritis

**DOI:** 10.1371/journal.pone.0183726

**Published:** 2017-08-24

**Authors:** Chien-Hsueh Tung, Ming-Chi Lu, Ning-Sheng Lai, Shu-Fen Wu

**Affiliations:** 1 Division of Allergy, Immunology and Rheumatology; Dalin Tzu Chi Hospital, Buddhist Tzu Chi Medical Foundation, Dalin, Chiayi, Taiwan, Republic of China; 2 School of Medicine, Tzu Chi University, Hualien, Taiwan, Republic of China; 3 Department of Life Science, Institute of Molecular Biology, National Chung-Cheng University, Min-Hsiung, Chia-Yi, Taiwan, Republic of China; Universita degli Studi di Napoli Federico II, ITALY

## Abstract

**Contexts:**

CD154 (commonly referred to as CD40-ligand) is a critical T cell factor that participates in the pathogenesis of autoimmune and is over-expressed in rheumatoid arthritis (RA). TNF-α blockade treatment had dramatic efficacy in RA.

**Objective:**

To investigate whether TNF-α blockade treatment can inhibit CD154 expression in RA.

**Methods:**

Blood samples were collected from 33 patients with rheumatoid arthritis before and 3 months after TNF-α blockade treatment. Clinical serological data determined by standard assays and T cell CD154 expression levels determined by flow cytometry were statistically analyzed for these two time points.

**Results:**

The percentage of CD154 expression on gated CD4+ T cells of PBMCs from RA patients after 3 months TNF-α blockade treatment was significantly lower than before treatment (2.94 ± 3.21% vs. 7.21 ± 5.64%; p = 0.0001). The disease activity and anti-CCP antibody levels were also significantly reduced after TNF-α blockade treatment. The CD154 expression levels were positively correlated with disease activity index DAS28, and CRP. The post-stimulated CD154 expression percentage of purified CD4+ T cells between baseline and after TNF-α blockade treatment was not significantly different (p = 0.221). Baseline CD154 levels were positively correlated with treatment-induced changes in DAS28 (p = 0.014; r2 = 0.187).

**Conclusions:**

TNF-α blockade treatment significantly decreased the CD154 expression on CD4+ T cells, disease activity and anti-CCP antibody simultaneously in RA patients. However TNF-α blockade did not impair T cell capacity to express CD154 after stimulation. These results suggest that decreased CD154 expression after TNF-α blockade may be due to decreased RA disease activity but not direct inhibition of CD154 responsiveness of T cells.

## Introduction

Rheumatoid arthritis (RA) is a chronic erosive polyarthritis of joint destruction with a poor prognosis. Many inflammatory cell subsets and proinflammatory cytokines participate in the pathogenesis. Joint pannus infiltration of monocytes, macrophages, B and T lymphocytes was found [1]. Disease modification treatment using conventional anti-rheumatic drugs provides limited protection [2–4]. Development of new pharmacological agents with targeted therapeutic actions is currently underway. Tumor necrosis factor-α (TNF-α) is the key cytokine in activation of the osteoclast and induction of bone erosion [5]. TNF-α antagonists, like etanercept, adalimumab, and infliximab, are already used clinically for treating rheumatoid arthritis with dramatic efficacy [2–4].

CD154 (commonly referred to as CD40-ligand, CD40L) is an important surface marker that participates in the immune response. It belongs to TNF superfamily [6,7]. It presents as clustering homotrimer complex on cell membrane surface and is found in multiple subsets of cells, mainly T lymphocytes. CD154 is transiently expressed on T cell surface after T cell activation. After binding with its ligand, CD40, it can promote T cell dependent B cell proliferation, maturation, antibody formation and immunoglobulin isotype switch. If mutation of CD154 occurred, hyper IgM syndrome with immunodeficiency happened. Hence it is critical in pathogenesis of Th2 cell mediated autoimmune disease, like systemic lupus erythematosus [8]. Besides, CD154 can also interact with CD40 on dendritic cells and monocyte/macrophage to promote cell differentiation, proinflammatory cytokine production and upregulated expression of costimulatory ligands. Hence, CD154 is also a crucial marker for Th1 cell mediated autoimmune disease, like rheumatoid arthritis [9,10].

Increased expression of CD154 on T cell surface was found in many autoimmune diseases, including systemic lupus erythematosus, Sjogren syndrome, ankylosing spondylitis, psoriasis and Behcet’s disease [8,10–12]. However, only mild increased expression of CD154 was found in rheumatoid arthritis[9]. Furthermore, a previous study demonstrated positive relationship between disease activity and CD154 expression level in rheumatoid arthritis [9]. Decreased CD154 expression after conventional disease modifying treatment was noted [9]. Besides, previous studies revealed that TNF-α blockade treatment decreased CD154 expression in ankylosing spondylitis and Crohn disease [12,13]. However, to date, it is not clear whether TNF-α blockade treatment modulated CD154 expression in rheumatoid arthritis.

Because CD154-CD40 signaling is associated with disease activity and pathogenesis of autoimmune diseases, in this study, we compared levels of CD154 expression before and after TNF-α blockade treatment in RA patients to evaluate whether TNF-α blockade had CD154 modulation effect and to investigate the possible underlying mechanisms.

## Patients and methods

### Patients

Thirty three patients (7 male and 26 females; mean age = 54.12 ± 11.28 yrs) with rheumatoid arthritis who fulfilled the revised RA criteria of the American College of Rheumatology were included in this study. All patients were receiving disease modifying anti-rheumatic drugs (DMARDs: hydroxychloroquine, methotrexate, leflunomide), low-dose prednisolone (<10 mg/day), and non-steroidal anti-inflammatory drugs (NSAIDs). None of these patients had received biological agents previously (Table 1, S1 Table).

**Table 1:** Demographic data of RA patients.

Demographic data of RA patients (n = 33)		
clinical characteristics		means ± SD
age (mean years ± s.d.)		54.12 ± 11.28
sex F/M		26: 7
duration (year)		5.48 ± 4.98
DAS 28 score		5.88 ± 0.85
medication		
	prednisolone (mg)	7.8 ± 4.79
	methotrexate	90.9%
	hydroxychloroquine	51.5%
	sulfasalazine	48.4%
	leflunomide	33.3%
	cyclosporine	6.0%
immunological level		
	RF-IgM (IU/ml)	201.6 ± 349.6
	anti-CCP (U/ml)	155.2 ± 144.8
	ESR (mm/hr)	29.2 ± 22.5
	CRP (mg/dl)	1.45 ± 1.47

Data are expressed as means ± SD. SD: standard deviation, DAS 28: RA disease activity Index, RF: rheumatoid factor, anti-CCP: anti-cyclic citrullinated peptide antibody, ESR: erythrocyte sedimentation rate, CRP: C-reactive protein.

All patients had severe peripheral joint disease and were administered TNF-α blockade treatment on schedule. All patients received etanercept treatment of 25mg twice weekly. Disease activity was assessed by evaluating the DAS28 (rheumatoid arthritis disease activity score), erythrocyte sedimentation rate (ESR) and C-reactive protein (CRP). Blood samples were collected at baseline and after 3 months of TNF-α blockade treatment. During the 3-month treatment period, the baseline drugs were not changed. The only additional drug was etanercept. None of the patients received steroids during the last 12 hours before blood collection.

The study protocol was approved by the Institutional Review Board of the Buddhist Tzu Chi General Hospital (BTZH IRB No.: B10302004) and written informed consent according to the Declaration of Helsinki was obtained from all of the participating individuals.

### Serological tests

Serum samples were obtained from all patients before and 3 months after TNF-α blockade treatment and stored at -80°C until analysis. A commercial second generation ELISA test was used for anti-CCP (Phadia GmbH, ImmunoCAP 100, Freiburg, Germany) according to the manufacturer’s instructions. The results of the anti-CCP test were considered positive if the antibody level was greater than the cut-off value of 10 U/ml. Rheumatoid factor (RF) was determined by laser nephelometry for the IgM isotype (Roche Diagnostic GmbH, Mannheim, Germany), and a level >14 IU/ml was considered positive. Acute phase reactants were determined by ESR (mm/h) and CRP (mg/dl) using standard laboratory methods.

### Medium, buffer and reagents

Cells were cultured in RPMI-1640-based medium (Gibco/BRL, Carlsbad, CA, USA) supplemented with 1% L-glutamine, 100 U/ml Penicillin, 100 U/ml streptomycin, 10% fetal bovine serum (Hyclone, Logan, UT, USA), 10mM HEPES, and 50μM β-mercaptoethanol. Phorbol 12-myrisate 13-acetate (PMA) (Sigma, St. Louis, MO, USA, P8139) was used at 50 ng/mL, while ionomycin (Sigma, I3909) was used at 1 μg/mL.

### Cell separation

Human peripheral blood mononuclear cells (PBMCs) were isolated from heparinized venous blood of RA patients using Ficoll-Hypaque density gradient centrifugation. In short, 20ml fresh blood was collected and spun at 2000 rpm for 10 min at RT. Blood plasma (upper fraction) was discarded, while blood cells in the lower fraction were resuspended in an equal volume of 1X PBS, followed by overlay onto a half volume of Ficoll-paque Plus (GE Healthcare, Buckinghamshire, United Kingdom, 17-1440-03). Cells were centrifuged at 2000 rpm for 20 min at RT, resulting in the generation of the PBMC fraction between serum and Ficoll-paque fractions for collection. Subsequently, PBMCs were resuspended in PBS/3% human IgG (Baxter International) to block Fc receptors and prevent non-specific Ab binding. Purified CD4+ T cells were isolated from PBMCs by CD4+ T cell isolation kits (STEMCELL Technologies, Vancouver, Canada, 19052) according to the manufacturer’s protocol.

### In vitro stimulation treatment of CD4+ T cells

CD4+ T cells isolated from blood of RA donors were diluted to a concentration of 1x10^**6**^/mL for experiments. The experiment cells, 2x10^**5**^ cells/ 200μl, were grown in 96-well tissue culture plates; then activated by stimulators of ionomycin (1 μg/mL) and PMA (50 ng/mL). Cells were harvested after stimulated for various durations as indicated and stained for CD4 or CD154, according to the manufacturer’s guidelines.

### Flow cytometric analysis

Flow cytometric analysis was performed using a Becton Dickinson FACS Calibur flow cytometer and CellQuest software (BD Biosciences). PBMCs were washed and subsequently incubated with saturating concentrations of the indicated antibody diluted in 200μl blocking buffer at 4°C for 20 minutes, spun, and then washed two times with cold PBS according to the manufacturer’s instructions. Cell staining for flow analysis was performed with the following specific antibodies: murine anti-human CD4 fluorescein isothiocyanate (anti-CD4-FITC) (Cat #555346) and anti-human CD154 phycoerythrin (anti-CD154-PE) (Cat #555700) conjugated monoclonal antibodies, purchased from BD Pharmingen, and their respective isotype control mAbs (BD Pharmingen).

### Statistical analysis

Student’s t-test was used to compare the means of two samples (before and after TNF-α blockade treatment); Mann-Whitney test was used to compare the means of two samples (health control vs RA patients or with vs without low disease activity). P-values <0.05 were considered statistically significant. Association between the percentage of CD154 expression and clinical parameters were evaluated by Pearson correlation and linear correlation analysis. A two-tailed significance level was set at p < 0.05. Statistical analysis used SPSS 10.0.

## Results

### TNF-α blockade treatment decreased CD154 expression of CD4+ T cells

**Fig 1:**
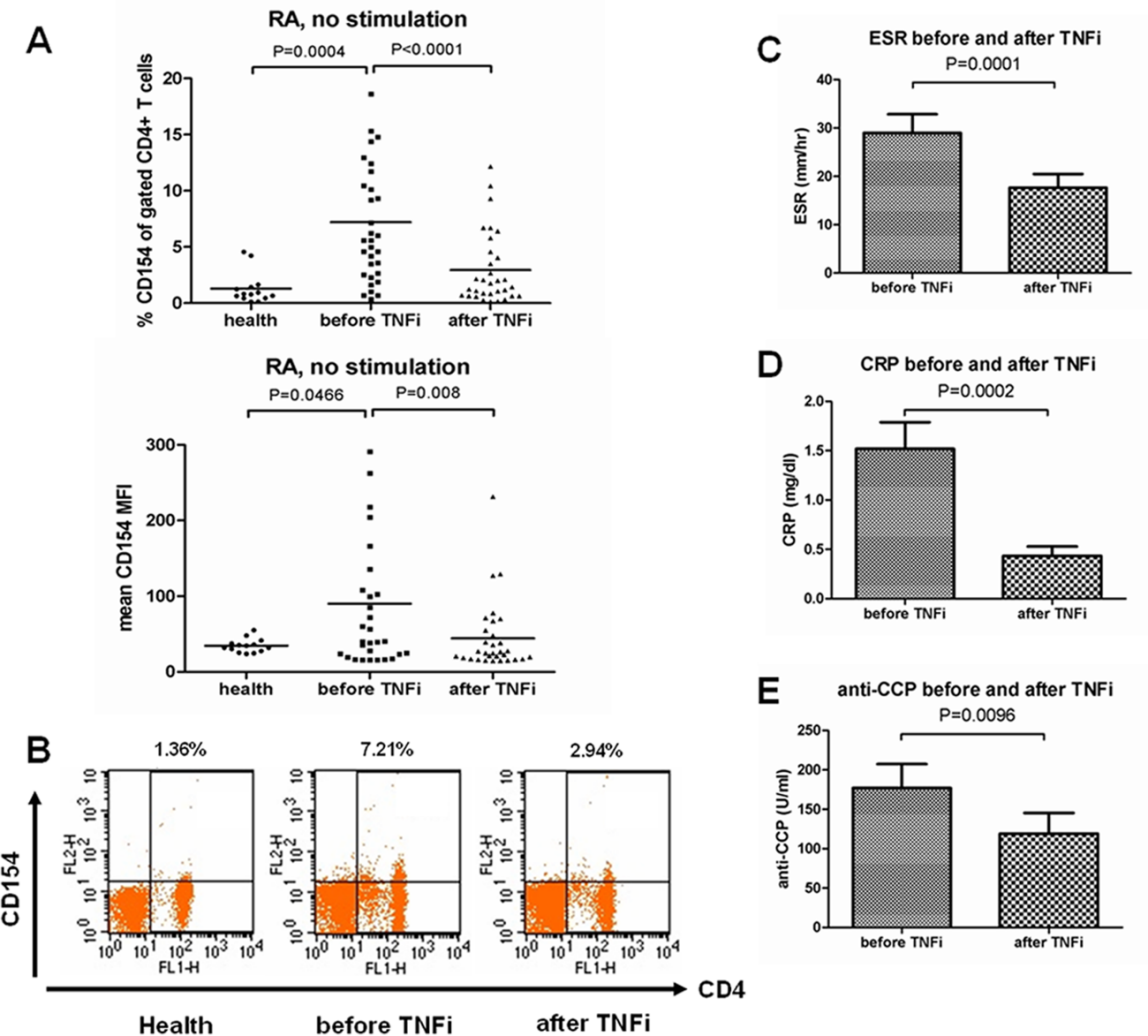
CD154 expression levels and clinical parameters in RA patients before and after TNF-α blockade treatment. (A) and (B) PBMCs were isolated from health control (n = 14) and RA patients (n = 33) before and after TNF-α blockade treatment for 3 months. The CD154 expression levels (CD154 percentage and MFI of gated CD4+ T cells) were measured by flow cytometry. Data were presented as means. Comparison of clinical parameters including ESR (C), CRP (D) and anti-CCP autoantibody (E) in RA patients before and after TNF-α blockade treatment. Data were presented as means ± SEM. TNFi: TNF-α inhibitor; MFI: mean fluorescence intensity; ESR: erythrocyte sedimentation rate; CRP: C-reactive protein; anti-CCP: anti-cyclic citrullinated peptide antibody.

TNF-α blockade treatment had dramatic effect on RA and decreased disease activity. Previous studies revealed that CD154 expression was associated with RA disease activity and decreased after treatment with traditional DMARD treatment [9]. To determine whether TNF-α blockade had any effect on CD154 expression, we isolated PBMCs from RA patients and investigated the percentages of CD154 on gated CD4+ T cells by flow cytometry at baseline and 3 months after TNF-α blockade treatment. The CD154 expression (as mean percentage or MFI) of gated CD4+ T cells in active RA patients before TNF-α blockade treatment was significantly higher than in healthy control (p = 0.0004 by percentage and p = 0.0466 by MFI) (Fig 1A, S1 Table). The CD154 expression levels were significantly attenuated after treatment (as mean percentage of gated CD4+ T cells: mean ±SD: 2.94 ± 3.12% versus 7.21 ± 5.64% at baseline, p < 0.0001) (Fig 1A and 1B, S1 Table). Besides, clinical disease activity, determined by ESR, CRP levels and DAS28, was also significantly reduced after TNF-α blockade (mean ±SD: 17.8 ± 16.6 versus 29.2 ± 22.5 at baseline, p = 0.0001; 0.44 ± 0.54 versus 1.45 ± 1.47 at baseline, p = 0.0002; 4.86 ± 1.16 versus 5.88 ± 0.85 at baseline, p = 0.0001; respectively) (Fig 1C and 1D, Table 2). Furthermore, the serum levels of anti-CCP antibodies were obviously reduced after TNF-α blockade treatment (mean ±SD: 129.4 ± 135.3 versus 155.2 ±144.8 at baseline, p = 0.009) (Fig 1E). However, although the serum levels of RF were also decreased after TNF-α blockade, the difference was not statistically significant (mean ±SD: 168.6 ± 274.7 versus 201.6 ± 249.6 at baseline, p = 0.125) (Table 2).

**Table 2:** CD154 expression and clinical parameters at baseline and after TNF-α blockade treatment.

	before TNFi treatment	after TNFi treatment	p value
% CD154/CD4	7.21 ± 5.64	2.94 ± 3.21	0.0001
DAS28 score	5.88 ± 0.85	4.86 ± 1.16	0.0001
ESR (mm/hr)	29.2 ± 22.5	17.8 ± 16.6	0.0001
CRP (mg/dl)	1.45 ± 1.47	0.44 ± 0.54	0.0002
anti-CCP (U/ml)	155.2 ± 144.8	129.4 ± 135.3	0.009
RF-IgM (IU/ml)	201.6 ± 249.6	168.6 ± 274.7	0.125
IgG (mg/dl)	1297.3 ± 397.8	1315 ± 391.1	0.82
IgA (mg/dl)	314.4 ± 145.8	321.1 ± 151	0.707
IgM (mg/dl)	112.5 ± 72.8	124 ± 76.8	0.06

Data are expressed as means ± SD. TNFi: TNF-α inhibitor, DAS 28: RA disease activity Index, ESR: erythrocyte sedimentation rate, CRP: C-reactive protein, RF: rheumatoid factor, anti-CCP: anti-cyclic citrullinated peptide antibody. Statistical significance was assessed by the paired t-test analysis. p values <0.05 were considered to be statistically significant.

### The CD154 expression was associated with disease activity

**Fig 2:**
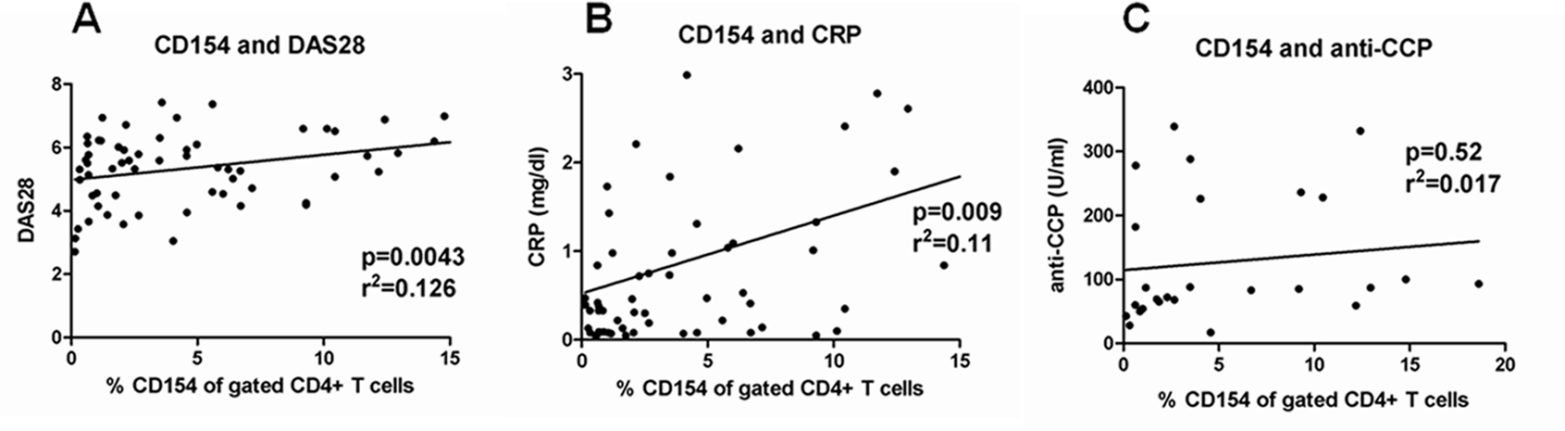
Association between the CD154 expression and the clinical parameters in RA patients. The correlation between the CD154 expression levels (% CD154 of gated CD4+ T cells measured by flow cytometry) and the levels of RA disease activity index DAS28 (A), inflammatory marker CRP (B) and anti-CCP autoantibody (C) in RA patients. CRP: C-reactive protein; anti-CCP: anti-cyclic citrullinated peptide antibody.

Our study revealed that CD154 expression decreased after TNF-α blockade treatment with concurrent decrease of RA disease activity. To verify the association between CD154 and clinical characteristics, Pearson and linear correlation assays were used for analysis. The levels of expressed CD154 were significantly associated with RA disease activity score DAS28 (p = 0.0043; r2 = 0.126) (Fig 2A) and inflammatory marker CRP (p = 0.009; r2 = 0.11) (Fig 2B). Furthermore, although the levels of CD154 expression and anti-CCP antibody both decreased after TNF-α blockade treatment, the correlation between CD154 and anti-CCP antibody levels was not significant (p = 0.52; r2 = 0.017) (Fig 2C). In addition, the CD154 expression levels did not correlate with ESR, RF-IgM, IgG, IgA and IgM (data not shown).

### The post-stimulated CD154 expression was not different after TNF-α blockade

**Fig 3:**
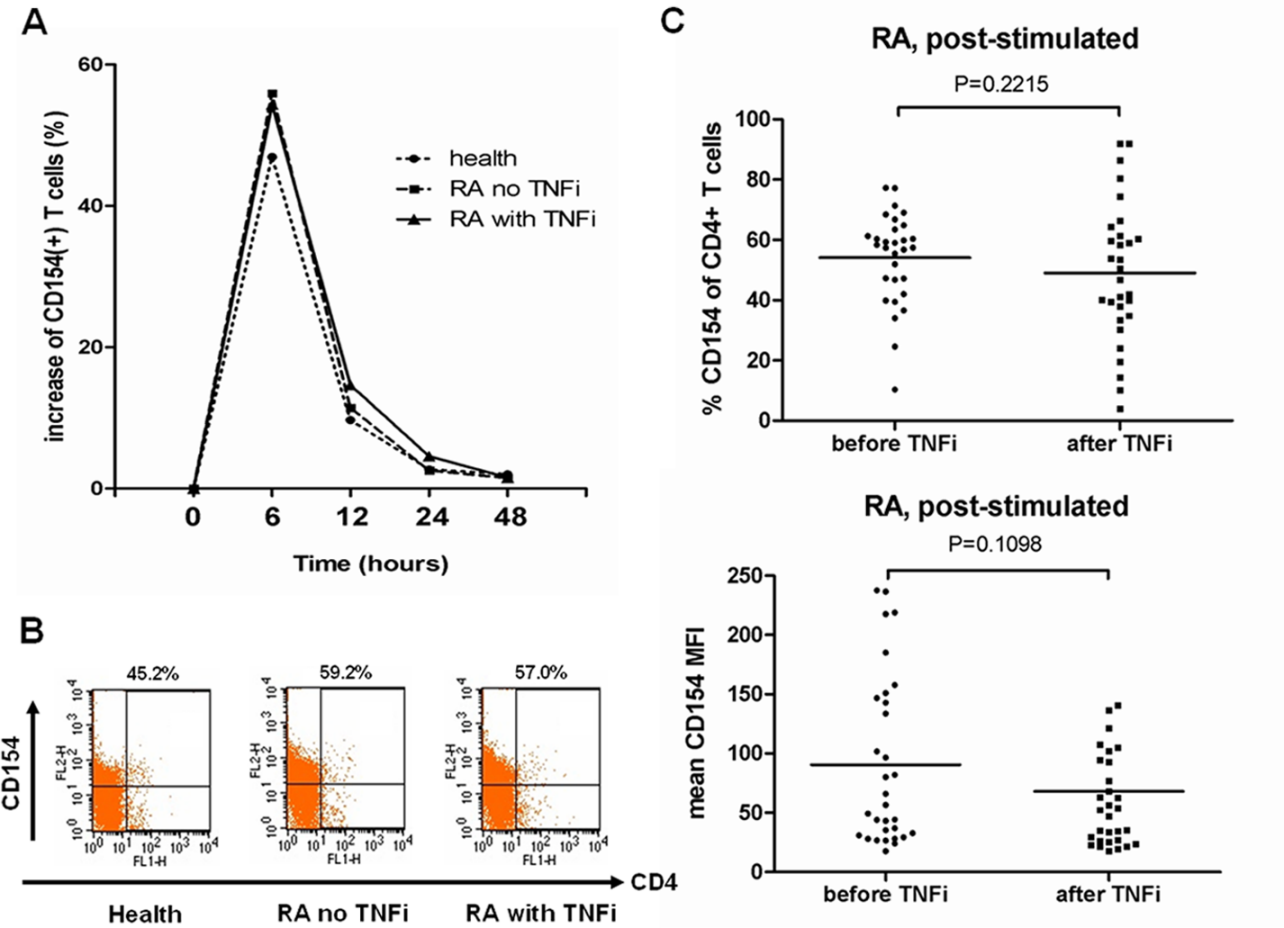
The post-stimulated CD154 expression levels in RA patients between baseline and after TNF-α blockade treatment. (A) and (B) CD4+ T cells were purified from health control (n = 7) and RA patients with (n = 8) and without (n = 9) TNF-α blockade treatment. After stimulation with ionomycin (1 μg/mL) and PMA (50 ng/mL) for different time periods, the CD154 expression levels (% CD154 of CD4+ T cells) were measured by flow cytometry. Data were presented as mean increase of CD154 percentage of CD4+ T cells. (C) CD4+ T cells were purified from RA patients (n = 30) before and after TNF-α blockade treatment for 3 months. After stimulation with ionomycin (1 μg/mL) and PMA (50 ng/mL) for 6 hours, the CD154 expression levels (CD154 percentage and MFI of CD4+ T cells) were measured by flow cytometry. Data were presented as mean percentage and MFI. TNFi: TNF-α inhibitor; MFI: mean fluorescence intensity.

In the previous study, after in vitro stimulation, T cells from RA patients have higher and longer CD154 expression than T cells from normal peripheral blood [14]. We performed similar experiments and investigated the kinetic change of CD154 expression after in vitro stimulation for different durations. We found the similar results (Fig 3A). The increased amplitude of CD154 expression was higher in RA patients than healthy control at 6 hours after stimulation (Fig 3A and 3B). After stimulation for 12 hours and 24 hours, the amplitude of CD154 level change in RA patients decreased but was still higher than in health control. At 48 hours, the expressed CD154 regained level similar to that before stimulation. In addition, the post-stimulated dynamic changes of CD154 expression were similar between RA patients with and without TNF blockade treatment (Fig 3A and 3B).

CD154 expression was transiently increased after T cell activation [7]. To determine whether the attenuated CD154 expression of T cells after TNF-α blockade was secondary to decreased disease activity or due to primary inhibitory effect of TNF-α blockade treatment on T cells, purified CD4+ T cells from RA patients were stimulated with ionomycin and PMA for 6 hours. CD154 expression levels of post-stimulated T cells (as mean percentage or MFI) were analyzed by flow cytometry. These analyses showed CD154 had no significant difference between baseline and after 3 months TNF-α blockade treatment (as mean percentage of CD4+ T cells: mean ±SD: 49.0 ± 23.0% versus 54.1 ± 15.1% at baseline, p = 0.2215) (Fig 3C, S1 Table).

### Higher baseline CD154 expression can predict better treatment response

**Fig 4:**
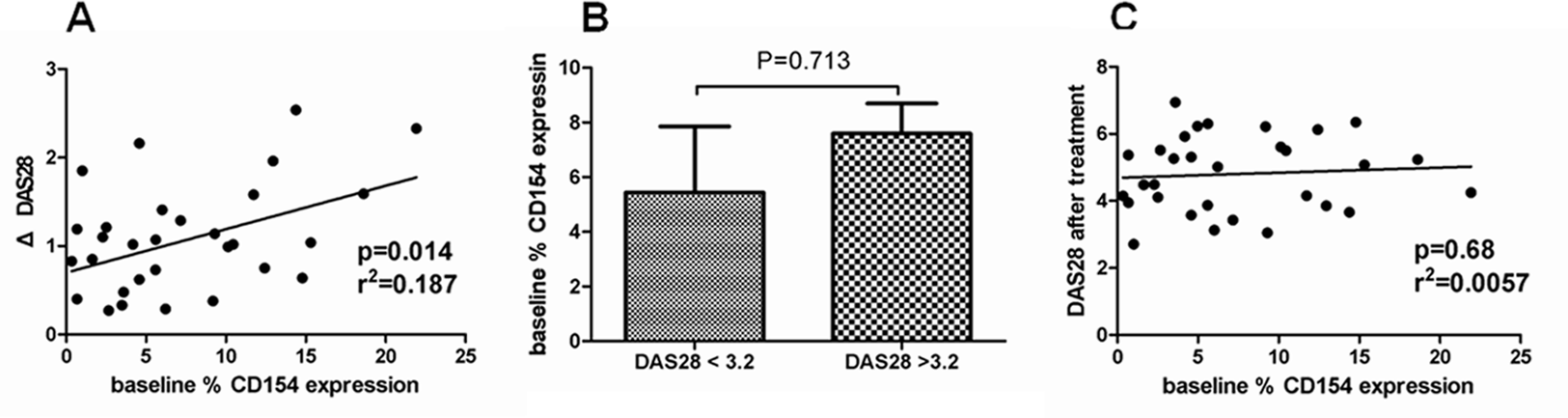
Relationship between baseline CD154 expression and clinical outcome after TNF-α blockade treatment in RA patients. (A) The correlation between the baseline CD154 expression levels (% CD154 of gated CD4+ T cells measured by flow cytometry) and the change of RA disease activity index DAS28 after TNF-α blockade treatment for 3 months. (B) Comparison of baseline CD154 expression levels between RA patients with and without low disease activity (DAS28 <3.2) after TNF-α blockade treatment. (C) The correlation between the baseline CD154 expression levels and the RA disease activity index DAS28 after 3-month TNF-α blockade treatment.

To investigate whether baseline CD154 can predict the treatment response, we evaluated the correlation between the baseline CD154 expression and the change of DAS28. We found that the baseline CD154 expression levels were positively correlated with treatment-induced DAS28 changes (p = 0.014; r2 = 0.187) (Fig 4A). This result indicated patients with higher baseline CD154 expression had better treatment response. The patients with lower basal CD154 expression had smaller change of disease activity after treatment.

Furthermore, we want to know whether baseline CD154 expression can be used to predict remission, low disease activity or relapse after TNF-α blockade treatment. There was no patient achieved remission status or experienced relapse in our study because shorter treatment duration and continuous usage of TNF-α blockade treatment. Therefore, we analyzed the baseline CD154 expression levels between the post-treatment patient with low disease activity (DAS28 <3.2) and those without low disease activity (DAS28 > 3.2). The baseline CD154 levels were lower in patients achieved low disease activity after treatment but not significant (p = 0.713) (Fig 4B). In addition, we evaluated the correlation between baseline CD154 expression and post-treatment DAS28. We found that there was no significant correlation (p = 0.68; r2 = 0.0057) (Fig 4C). Therefore, the baseline CD154 can’t be used to predict the low disease activity and post-treatment DAS28 after 3-months TNF-α blockade treatment.

## Discussion

Rheumatoid arthritis is a chronic destructive autoimmune disease. CD154-CD40 signaling has a critical role in pathogenesis of autoimmune diseases [7,10]. Enhanced CD154 expression was found on peripheral blood T cells and synovial tissue in RA patients [9,15]. To date, TNF-α blockade treatment was commonly used in RA and had obvious efficacy [2–4]. Our results demonstrated that TNF-α blockade treatment for 3 months could decrease the expressed frequency of CD154+CD4+ T cells in RA patients (Fig 1A).

Because CD154 expression is transiently induced on cell surface after T cell activation, lower CD154 expression may represent lower T cell activation [7,16]. Our results demonstrated that the expressed CD154 level was positively correlated with disease activity index DAS28 (Fig 2A). Reduced RA disease activity after TNF-α blockade treatment may cause fewer T cell activation and subsequently lower CD154 expression. Furthermore, CD154 expression levels of post-stimulated T cells from RA patients receiving 3 months TNF blockade treatment were not different from baseline (Fig 3C). The capacity of T cells to express CD154 was not impaired after TNF-α blockade. Therefore, we concluded that the lower expression of CD154 on T cells after TNF-α blockade may be secondary to decreased disease activity and a loss of micro-environmental inflammatory stimulation, but not due to primary inhibitory effect of TNF-α blockade on T cell capacity to express CD154. TNF-α blockade did not impair T cell CD154 responsiveness, and higher CD154 expression could be induced after ex vivo stimulation (Fig 3C).

A previous study revealed CD154 expression was higher and longer in RA patients than in health control [14]. CD154 expression is dependent on Ca^**2+**^–NFAT signaling and IL-15 –STAT5 signaling pathway [17–19]. Increased Ca^**2+**^ influx as well as increased Ca^**2+**^ release-activated Ca^**2+**^ channel protein expression and function was noted in RA patients [20]. Besides, increased IL-15 level in serum and synovial fluid of RA patients was found [21,22]. Thus, RA patients had higher CD154 expressed T cells than health control. The short-term calcium influx response after T cell activation can be inhibited after TNF blockade treatment in RA patients [23]. In addition, treatment with TNF blockade in RA patients can reduce the serum levels of IL-15 [24]. Therefore, in our study, the decrease in CD154 expression after TNF blockade may be due to a decrease in calcium response and serum IL-15 after treatment.

The previous studies showed that CD154 expression was higher in RA patients with higher disease activity [9,14]. This was consistent with our results showing that that CD154 expression was positively correlated with disease activity and inflammatory marker CRP. An anti-inflammatory effect of TNF-α blockade with decreased pro-inflammatory cytokines, such as IL-1, IL-6, IL-8 and IL-15, has been proposed [24–26]. After TNF-α blockade, lower extrinsic cytokine stimulation to T cells may induce lower CD154 expression. Furthermore, TNF-α itself could costimulate TCR mediated T cell activation and thereby augment T cell proliferation, expression of activation marker and cytokine secretion [27,28]. One study in patients with Crohn disease revealed that TNF-α blockade (infliximab) could directly attenuate post-stimulated T cell CD154 expression in vitro and promote T cell apoptosis [13]. Therefore the TNF-α blockade may directly reduce T cell capacity of CD154 expression in patients with Crohn disease. However in our ex vivo study, the capacity of CD154 expression in T cells was not inhibited in RA patients receiving TNF blockade treatment (etanercept). The difference may be due to different disease groups and treatment biologics. In addition, TNF-α could diminish Foxp3 synthesis and inhibit suppressive function of regulatory T cells [29]. TNF-α blockade treatment could induce a distinct population of CD62L- regulatory T cells and restore the defective suppressive capacity of CD4+CD25^**high**^ regulatory T cells in RA [29,30]. Regulatory T cell had an essential role in maintain homeostasis and suppress autoimmune [31]. The regulatory T cells could attenuate effector T cell proliferation and activation. Therefore, those extrinsic anti-inflammatory processes of TNF-α blockade may cause lower CD154 expression in our RA patients without impairing T cell capacity to express CD154 after stimulation ex vivo.

Rheumatoid arthritis specific autoantibody had been showed having a pathogenic role and increased TNF-α synthesis from macrophage in dose dependent manner [32,33]. The presence of anti-CCP antibody is associated with higher disease severity and radiological erosion [34,35]. It is evident that after TNF-α blockade treatment, the levels of anti-CCP antibodies decrease [35,36]. Consisting with our study, the serum titers of anti-CCP antibodies were significantly reduced after TNF-α blockade for 3 months. To date, the underlying mechanisms that anti-CCP antibody levels decreased after TNF-α blockade treatment were unclear. CD154 was a critical factor for antibody synthesis. CD40-CD154 signaling was important for the production of anti-CCP antibodies by peripheral blood B cells in vitro [37]. In our study, the CD154 and anti-CCP antibody level both decreased after TNF-α blockade treatment (Fig 1A and 1D). However, we investigated the correlation between the levels of CD154 expression and the anti-CCP antibody levels and found no significance (Fig 2C). This result may be due to that only 19 patients in our study had positive anti-CCP antibody and we excluded the patients with anti-CCP antibody >340 U/ml because 340 U/ml is the highest concentration we can test for anti-CCP antibody. More advanced test methods and larger study population may help us to assess the correlation in the future.

The previous study found that CD154 expression decreased after conventional DMARD treatment [9]. In our study, the CD154 levels were positively correlated with RA disease activity (Fig 2A). The CD154 expression can be induced after stimulation in T cells from RA patients under TNF-α blockade treatment (Fig 3C). Therefore down modulation of CD154 was not specifically related to TNF-α blockade treatment and was related to decreased disease activity. Higher CD154 expression in RA patients may indicate the higher activity and resistance to current therapy.

In the previous study, change of CD154 expression after treatment can be used to predict x-ray progression in RA patients [38]. Besides, higher CD154 expression could predict clinical efficacy of spondyloarthropathy patients with TNF-α blockade treatment [39]. In our study, higher baseline CD154 expression can predict better TNF-α blockade treatment response (Fig 4A). However, the baseline CD154 can’t be used to predict the low disease activity in 3-months TNF-α blockade treatment (Fig 4B). The post-treatment patients with low disease activity had lower baseline CD154 levels but not significant. These results may be due to short treatment duration in our study and only 3 patients achieved low disease activity status. The CD154 expression was positively correlated with RA disease activity (Fig 2A). Therefore, the patients with lower CD154 expression may be more likely to achieve remission and those with relapse may have higher CD154 levels comparing with that before relapse. Previous studies revealed RA patients with higher disease activity had better response to TNF-α blockade treatment [40]. Hence, higher baseline CD154 levels in RA patients indicated higher disease activity and better response to treatment in our study.

In conclusion, TNF-α blockade treatment significantly decreased the expression of CD154 on CD4+ T cells and attenuated clinical disease activity and anti-CCP antibody in RA patients. However, CD154 expression of post-stimulated T cell was similar, irrespective of TNF-α blockade. These results suggest that TNF-α blockade is likely to play an immune modulating role on CD154 expression through reducing external proinflammatory stimulation and T cell activation.

## Supporting information

S1 Table Demographic data and laboratory results. (XLS)
